# Simvastatin protects bladder and renal functions following spinal cord injury in rats

**DOI:** 10.1186/1476-9255-7-17

**Published:** 2010-04-19

**Authors:** Anandakumar Shunmugavel, Mushfiquddin Khan, Peter C te Chou, Ramanpreet K Dhindsa, Marcus M Martin, Anne G Copay, Brian R Subach, Thomas C Schuler, Mehmet Bilgen, John K Orak, Inderjit Singh

**Affiliations:** 1 Department of Pediatrics, Medical University of South Carolina, Charleston, SC, USA; 2 Department of Radiology, Medical University of South Carolina, Charleston, SC, USA; 3 The Spinal Research Foundation, Reston, VA, USA

## Abstract

**Background:**

Urinary bladder and renal dysfunction are secondary events associated with spinal cord injury (SCI) in humans. These secondary events not only compromise quality of life but also delay overall recovery from SCI pathophysiology. Furthermore, in experimental models the effects of SCI therapy on bladder and renal functions are generally not evaluated. In this study, we tested whether simvastatin improves bladder and renal functions in a rat model of experimental SCI.

**Methods:**

SCI was induced by controlled contusion of T9-T10 in adult female rats. Simvastatin (5 mg/Kg body weight) was administered at two hours after SCI and repeated every 24 hours until the end point. Simvastatin-treated SCI animals (simvastatin group) were compared with vehicle-treated SCI animals (vehicle group) in terms of the Basso Beattie Bresnahan score, tissue morphology, cell death, and bladder/renal functions.

**Results:**

The urinary bladder of vehicle animals showed a 4.3-fold increase in size and a 9-fold increase in wet weight compared to sham animals. Following SCI, the urine to plasma osmolality ratio increased initially but decreased 1 week after SCI. Hematoxylin and eosin staining of bladder tissue showed transitional epithelial hyperplasia, degeneration of lamina propria, and enlargement of tunica adventia in addition to detrusor muscle hypertrophy. Rats treated with simvastatin for 14 days displayed remarkable recovery by showing decreased bladder size and maintenance of a normal urine/plasma osmolality ratio, in addition to improved locomotion. The muscularis layer of the bladder also regained its compact nature in simvastatin animals. Moreover, SCI-induced renal caspase-3 activity was significantly decreased in the simvastatin group indicating the ability of simvastatin to reduce the renal tubular apoptosis.

**Conclusion:**

Post-injury administration of simvastatin ameliorates bladder and renal dysfunction associated with SCI in rats.

## Introduction

Spinal cord injury (SCI) results primarily in the loss of motor and sensory functions. Severe SCI often results not only in paralysis but also in the loss of sensation and reflexes below the point of injury, such as bowel and bladder control. Dysfunction of the urinary system is one of the most important consequences of SCI. Bladder dysfunction causes hyperarousal, sleep disturbances, and disruption of sensorimotor integration. Spinal and supraspinal circuitry controls urine storage and release [1,2]. Other brainstem nuclei like the raphe magnus, raphe pallidus, parapyramidal medullary reticular formation, subcoeruleus pars alpha, locus coeruleus and A5 and A7 nuclei are also involved in the bladder external urethral sphincter (EUS) pathway [3]. Signals from the pons project directly to the S2-S4 sacral segments of the spinal cord and control the detrusor and urethral sphincter activity parasympathetically, resulting in normal storage and voiding [4]. Detrusor-sphincter dyssynergia has been reported in SCI patients [5].

Understanding of bladder dysfunction comes from bladder outlet obstruction (BOO) [6-8] and experimental autoimmune encephalomyelitis (EAE) animal models [2]. Although bladder hypertrophy following SCI was reported previously [9], the bladder pathophysiology associated with SCI has not been thoroughly investigated. Since tissue hypertrophy depends on hypertrophying signals [10], investigating the pathophysiology of bladder dysfunction in SCI patients is significant. We investigated the pathophysiology of spinal bladder dysfunction in a rat contusion model of SCI. Contusion injuries are created by hitting the exposed spinal cord with a mechanical device that displaces the spinal cord by a preselected amount. The contusion injury model seems to be the most relevant to human spinal cord traumatic injury [11].

Current therapeutics for neurogenic bladder include functional electrical stimulation (FES) [12]; however, exposure to uncontrolled shock has been reported with delayed recovery to normal bladder function [13]. Other available treatment options include anticholinergics, self catheterization, and use of desmopressin, cannabinoids, vanilloids, and botulinum neurotoxin [14]. A bladder acellular matrix graft (BAMG) has also been shown to improve voiding function of SCI-induced hypertrophic bladder [15]. Despite the availability of various treatment options, catheterization is the most widely used technique in managing bladder problems with SCI patients. However, indwelling urethral catheterization is associated with complications like bladder stone formation or infection and causes significant morbidity [16]. Hence, catheter-free bladder maintenance is the ultimate aim of studies involving urogenital problems associated with SCI [17]. Furthermore, the direct effect of SCI therapies, including statins, on bladder and renal functions is not known. Therefore, this study investigates the therapeutic efficacy of simvastatin for restoring bladder and renal functions following SCI.

Statins are FDA-approved cholesterol lowering drugs widely used in clinical practice. Studies from our laboratory described anti-inflammatory properties of statins in a cell culture model [18]. Subsequently, studies from our laboratory and others have reported the immunomodulatory [19,20] and neuroprotective activities of statins in animal models of EAE [21,22]. These pleiotropic effects of statins were reported to function independently of their cholesterol lowering effects. Statins have also been reported to ameliorate the risk associated with metabolic syndrome in vascular and chronic kidney disease [23] as well as renal inflammatory diseases [24-27]. We observed neuroprotective and anti-inflammatory activities of atorvastatin in a post-injury treatment rat model of experimental SCI [28]. Since neurogenic bladder and renal dysfunction are associated with SCI and statin treatment has been reported to protect against SCI, we investigated the efficacy of simvastatin for bladder hypertrophy and renal dysfunction in a rat model of SCI.

## Methods

### Materials

Unless otherwise stated, all compounds were purchased from Sigma-Aldrich (St. Louis, MO, USA). Caspase 3 antibody (rabbit polyclonal) was from Santa Cruz, CA, USA. Simvastatin was purchased from Calbiochem, CA, USA.

### Animal

The animals used in the present study were female Sprague-Dawley rats (225-250 g) purchased from Harlan laboratories (Durham, NC). The animal procedures for the study were approved by the Institutional Animal Care and Use Committee (IACUC) of the Medical University of South Carolina.

### Experimental design and administration of simvastatin

The experiment consisted of three groups of animals: sham operated (sham), vehicle-treated (vehicle), and simvastatin-treated (simvastatin). Simvastatin (5 mg/kg in 1% methyl cellulose solution) was gavage fed to the animals at 2 hours after SCI and every 24 hours thereafter until the animals were sacrificed. Vehicle and sham animals were fed with carrier solution alone. Selection of 5 mg/kg dose of simvastatin is based on our earlier study on atorvastatin-mediated neurovascular protection following SCI in rats [28].

### Controlled contusion spinal cord injury

Animals were anesthetized with ketamine-xylazine cocktail (80 mg/kg-10 mg/kg body weight respectively). After confirming the validity of anesthesia by toe pinching, the animals were depilated on dorsal spine line, and a hemilaminectomy was done at the T9-T10 level to expose the dura overlying the spinal cord [29]. Spinal cord contusion injury was induced by a controlled contusion injury (CCI) device described by Bilgen [30]. Injury was made with 2 mm diameter impactor at 1.5 m/s velocity to a depth of 1 mm for 85 msec. Following SCI, the wound was irrigated with phosphate buffered saline (PBS) solution, and the incision was closed in layers, with the skin closed using polysorb 4. Sham-operated animals underwent laminectomy only. Animals were returned to their cages and kept on a 37°C heating blanket overnight.

### Evaluation of locomotor function

The locomotor activities of rats were recorded for 28 days according to the Basso Beattie Bresnahan (BBB) open field expanded locomotor rating scale [31]. The BBB rating was described with a 21-point scale to measure hind limb function at various time points after injury. The scale assesses 10 different categories, including limb movement and tail position. Sham operated animals scored 21 on the BBB scale, whereas the SCI animals with complete hind limb paralysis scored 0. Experimental animals were tested after SCI on days 3, 7, 14, 21, and 28. Each group consisted of at least 6 animals. Evaluations were made by two investigators blinded to the experimental groups.

### Bladder analyses

Bladders of experimental animals were emptied by gentle abdominal massage. At indicated times following SCI, the animals were sacrificed with intra peritoneal injections of sodium pentobarbital. After proper bladder emptying, they were perfused with PBS followed by neutral formalin solution. The bladders were extracted, weighed and preserved in 10% formalin (Fisher, Pittsburgh, PA, USA) for further fixation. Images of bladders were scanned, and the area of the images was calculated using BioRad Quantity One 4.6.5 image analysis software.

### Urine analyses

Animals were given abdominal massage to empty the bladder three times a day at 8.00, 13.00 and 20.00 hrs regularly, and the 12 h urine was used to measure volume, osmolality, and protein level. Osmolality was measured with a Microsmette (freezing point depression osmometry) instrument per the instructions of the manufacturer (Precision systems Inc, MA, USA). Each sample was measured in triplicate. Urine protein concentration was measured by the Bradford protein assay method. Plasma collected from 100 μl blood from the caudal vein was also analyzed in the same way.

### Histology

At designated time points, the animals were anesthetized with intra peritoneal injections of sodium pentobarbital and then transcardially perfused with PBS followed by neutral formalin solution. Bladder and kidney tissues were removed and fixed in 10% formalin solution. After fixation, the tissues were processed following routine histological procedures. Tissues were dehydrated in series of alcohol and infiltrated with paraffin wax (M.P 60°C) using Leica TP-1020 automatic tissue processor. Tissue blocks were sectioned (8 μm) with Leica HM-325 rotary microtome. Sections were adhered on to super frost plus gold slides (Fisher Scientific Inc, MA, USA). After suitable drying time, the sections were deparaffinized in 2 changes of xylene for 10 minutes each. Sections were rehydrated by passing through decreasing grades of ethanol (100, 95, 80, 70, and 30%) and water. Sections for morphological studies were processed and stained with hematoxylin and eosin (H&E) as described previously [32].

### Immunofluorescence study

Deparaffinized and rehydrated slides were boiled in antigen unmasking solution (Vector Labs, Burlingame, CA) for 10 min, cooled for 20 min, and washed with Tris-sodium buffer (0.1 M Tris-HCl, pH-7.4, and 0.15 M NaCl) with 0.05% Tween 20 (TNT) three times each for 5 min. The sections were then treated with trypsin (0.1% for 10 min). Endogenous peroxidase activity was eliminated by treating the section with 3% hydrogen peroxide solution for 10 min. Sections were blocked in TNT buffer with 0.5% blocking reagent (TNB, supplied with TSA-Direct kit; NEN Life Sciences, Boston MA) for 30 min to reduce nonspecific staining. The sections were incubated overnight with anti caspase-3 antibody (Santa Cruz, CA, USA; 1:200) at 4°C. After washing with PBS, the sections were stained with Alexafluor 488 (Molecular Probes, Invitrogen, CA, USA) flurophore conjugated secondary antibody. The tissue fluorescence pattern was observed and recorded with a Leica TCS SP5 Laser scanning microscope.

### Statistical analysis

Statistical analysis was performed by student *t *test using Graph pad -software. Data are expressed as mean ± standard deviation (SD). P < 0.05 was considered statistically significant.

## Results

### Simvastatin improves locomotor behavior in SCI rats

SCI is associated with apoptotic neuronal loss resulting in compromised locomotor functions. We confirmed the severity and consistency of SCI by the paraplegic outcome of the experimental animals. The effect of simvastatin in restoring locomotor behavior was evaluated by BBB score after SCI. The sham animals consistently scored 21 on the BBB locomotor scale. The vehicle treated group scored 0.57 ± 0.2 (day 3), 2.78 ± 0.93 (day 7), 6.08 ± 0.39 (day 14), 6.33 ± 0.35 (day 21), and 6.417 ± 0.41 (day 28) post SCI. The simvastatin group scored significantly higher (1.71 ± 0.612, 5.583 ± 0.69, 9.33 ± 0.9, 9.92 ± 1.2, and 10.58 ± 1.3 on day 3, 7, 14, 21, and 28 post SCI, respectively) than the vehicle group animals.

### Simvastatin reduces the size and weight of urinary bladder in SCI rats

**Figure 1:**
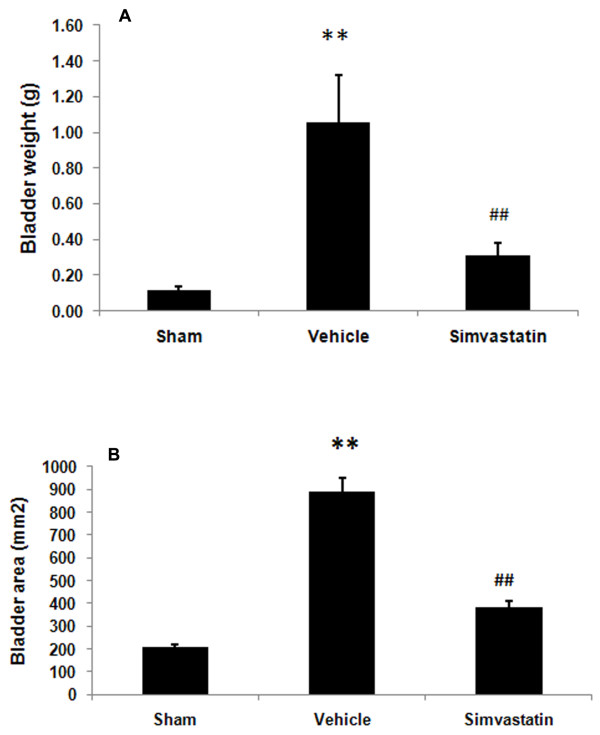
**Simvastain decreases weight and area of bladder after SCI in rats**. Wet weight (A) and area (B) of bladder of sham, vehicle and simvastatin groups were determined at 14 days after SCI. Data are expressed as mean ± SD, n = 7. **p < 0.01 vs. sham, ## p < 0.01 vs. vehicle.

**Figure 2:**
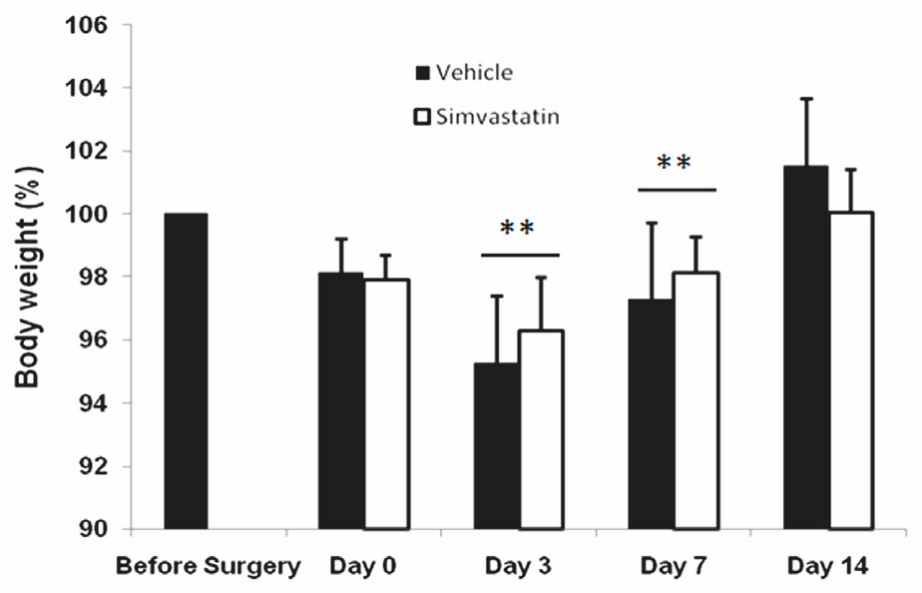
**Effect of simvastatin on body weight of rats after SCI**. Animal body weight after SCI was reduced significantly in both vehicle and simvastatin groups from day 3 to day 7. However, the weight was normal at day 14 after SCI in both of the groups. Results are presented as % change body weight and data are expressed as mean ± SD, n = 7. **p < 0.001 vs. before surgery.

Bladder size and weight changed significantly in animals with SCI (Fig 1). The bladder weighed 0.115 ± 0.22 g in the sham animals, whereas it was 1.05 ± 0.26 g in SCI animals, and the increase grew to 9-fold on day 14 after SCI. The simvastatin treated group of animals had only a 2.7 fold increase in bladder weight compared to the sham animals (Fig 1A). Bladder volume in terms of area also significantly increased in the vehicle group (888.582 ± 12.42 mm^2^). The increase in bladder volume compared to the sham group was 4.29-fold. The simvastatin-treated group had only a 1.85-fold increase in bladder area compared to the sham group (Fig 1B). In contrast, no significant difference in body weight loss between vehicle and simvastatin groups was observed during the experimental period (Fig 2).

### Simvastatin ameliorates voided volume, osmolality, and protein level of urine in SCI rats

**Figure 3:**
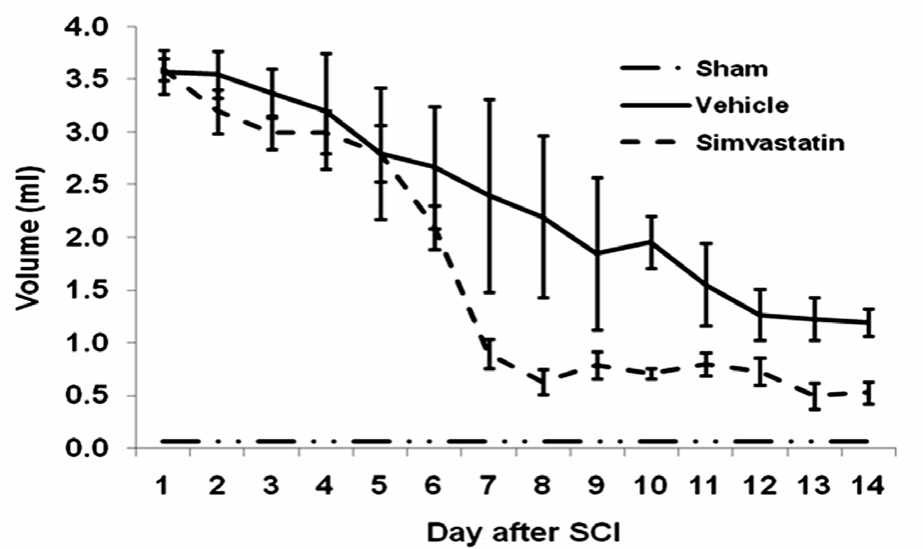
**Simvastatin reduces the voided urine volume after SCI in rats**. On day 1 post SCI, urine volume was 3.57 ± 0.21 ml in 12 hours. In vehicle group, the volume of urine retained in the bladder gradually decreased to 1.2 ± 0.13 ml on day 14. Simvastatin treated animals retained significantly lesser volume in the bladder from day 8 to 14. Sham animals retained less than 0.07 ml urine on all the days of experiment. Data are expressed as mean ± SD, n = 7.

**Figure 4:**
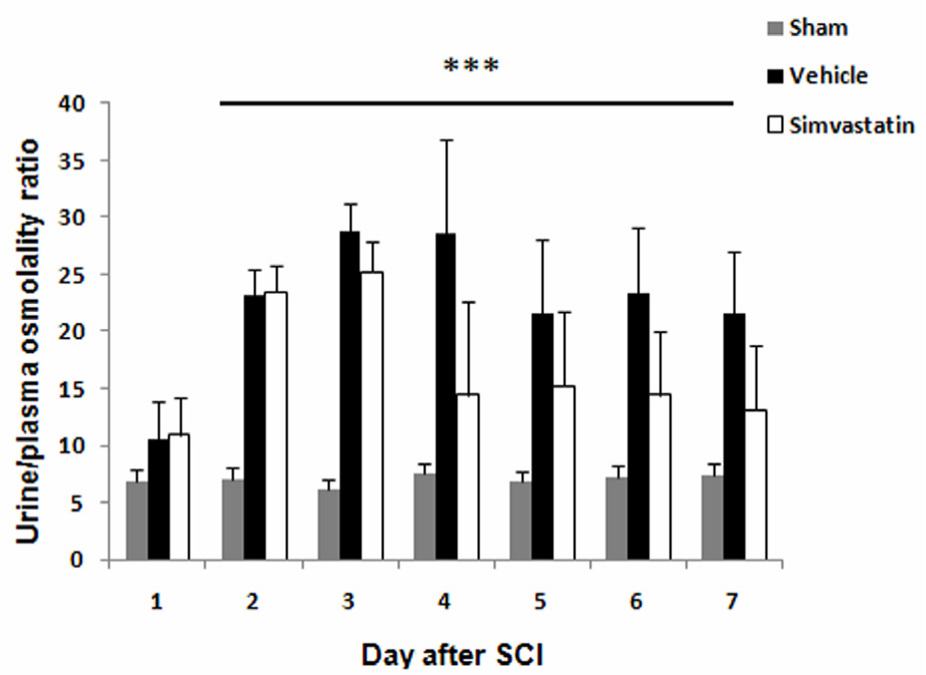
**Simvastatin reduces urine/plasma osmolality ratio after SCI in rats**. The ratio of urine/plasma osmolality in vehicle group was significantly higher than the sham and simvastatin groups from day 3 onward. Data are expressed as mean ± SD, n = 6. ***p < 0.001.

**Figure 5:**
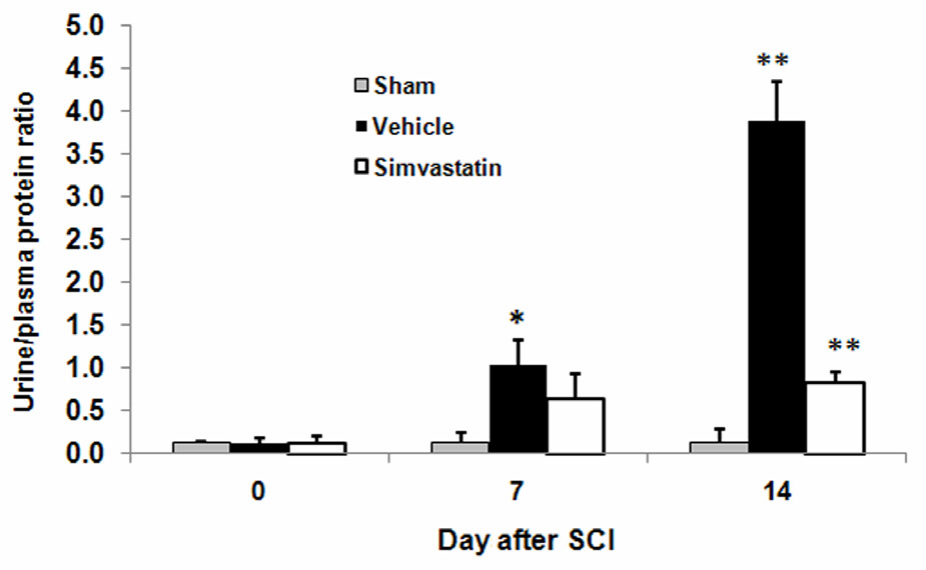
**Simvastatin ameliorates proteinuria after SCI in rats**. Urine/plasma protein ratio in sham, vehicle and simvastatin groups was determined as described in Methods. The ratio was significantly reduced in the simvastatin group (0.84 ± 0.16 compared to vehicle group (3.89 ± 0.45) on ^14^th day after SCI. Sham group did not show any change in the ratio. Results are expressed as ratio of urine/plasma proteins and data are expressed as mean ± SD, (n = 6).

The volume of voided urine in the bladder gradually decreased from day 1 to day 14 in SCI rats (Fig 3). The sham animals retained less than 70 μl of urine. Vehicle animals retained 1.2 ± 0.13 ml on day 14 after SCI, whereas simvastatin animals retained a significantly lower volume of urine (0.53 ± 0.01 ml) in the bladder. The change in urine to plasma osmolality ratio associated with SCI is given in Fig 4. Sham animals showed a ratio of 6.1 to 7.5 during the study period. The ratio ranged from 3.6 to 38.6 in the vehicle group. Vehicle animals showed a significant increase in the ratio. The simvastatin group of animals showed increasingly high urine/plasma osmolality ratios for 3 days after SCI. Later, the ratio decreased gradually and maintained the levels observed in sham animals. The quantity of protein excreted through urine was significantly elevated in vehicle animals. (Fig 5). The urine/plasma protein ratio was 0.124 ± 0.03 in sham animals. In the vehicle animal, the ratio increased to 1.03 ± 0.3 and 3.887 ± 0.45 on day 7 and 14, respectively. The simvastatin group showed significantly lower levels of protein excreted in urine on day 7 as well as day 14 after SCI.

### Simvastatin improves SCI-induced bladder and renal histopathology in SCI rats

**Figure 6:**
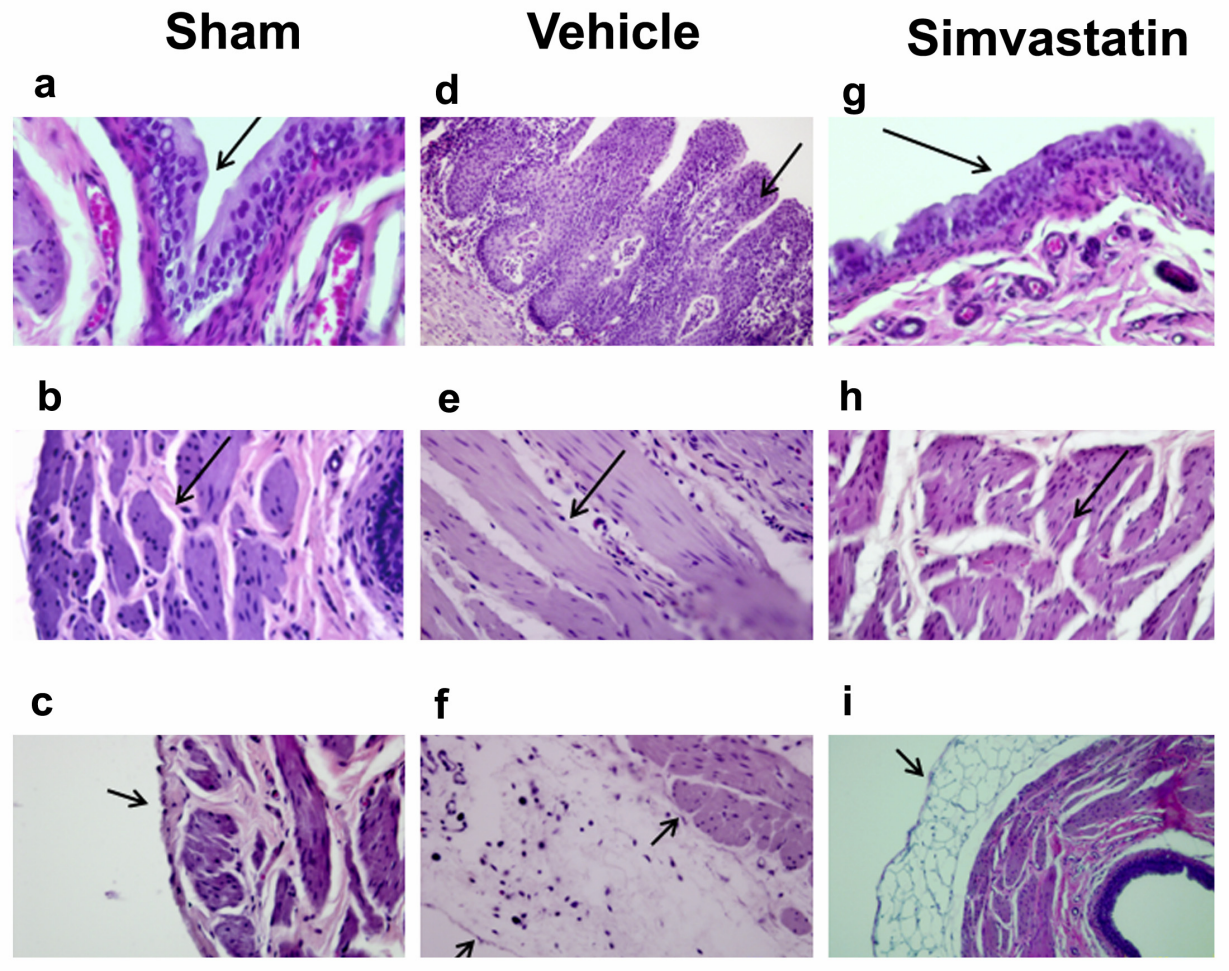
**Simvastatin improves histomorphology of spinal bladder evaluated at 14th day after SCI in rats**. Histomorphology of spinal bladder of sham (a-c), vehicle (d-f) and simvastatin group (g-i) was determined by H & E staining. The transitional epithelium was 3 cell layers thick in sham and covered with membrane plaque (a; arrow). Hyperplasia and degeneration of epithelial layer and degeneration of lamina propria were observed in spinal bladder (d). Muscle layers were thick with little matrix in sham control (b) but degenerated with large matrix deposition in spinal bladder (e; arrow). Tunica adventia enclosed large space filled with matrix in spinal bladder (f). Simvastatin treatment significantly decreased the transitional epithelial hyperplasia (g) muscular hypertrophy (h) and matrix in tunica adventia (i; arrow). Photomicrographs are representative of n = 3 in each group. Magnification × 400.

In the sham animal bladder, the transitional epithelial layer was about 3 cell layers thick and was covered with membrane plaque as shown in Fig 6a. The muscular layer was thick and compact (Fig 6b). Tunica adventia was thin and closely applied to the outer circular muscle layer of the bladder (Fig 6c). In the hypertrophied bladder of the vehicle treated animals, the transitional epithelial cells were several layers thick due to hyperplasia and also lacked proper organization (Fig 6d). Lamina propria was highly degenerated and was characterized by infiltration of cells (Fig 6e). The muscularis layer was highly disorganized, and the number of nuclei per muscle area was also decreased. The hypertrophied bladder was also characterized by thick and disorganized tunica adventia, and cellular infiltration was evident (Fig 6f). Simvastatin treatment showed remarkable recovery in terms of a reduced number of transitional epithelial cell layers and diminished infiltration of cells (Fig 6g). The muscularis layer was also compact (Fig 6h), while the tunica adventia thickening and disorganization observed in the vehicle treated group was significantly reduced in the simvastatin group (Fig 6i).

### Simvastatin improves tissue structure and reduces the expression of caspase-3 in kidney from SCI rats

**Figure 7:**
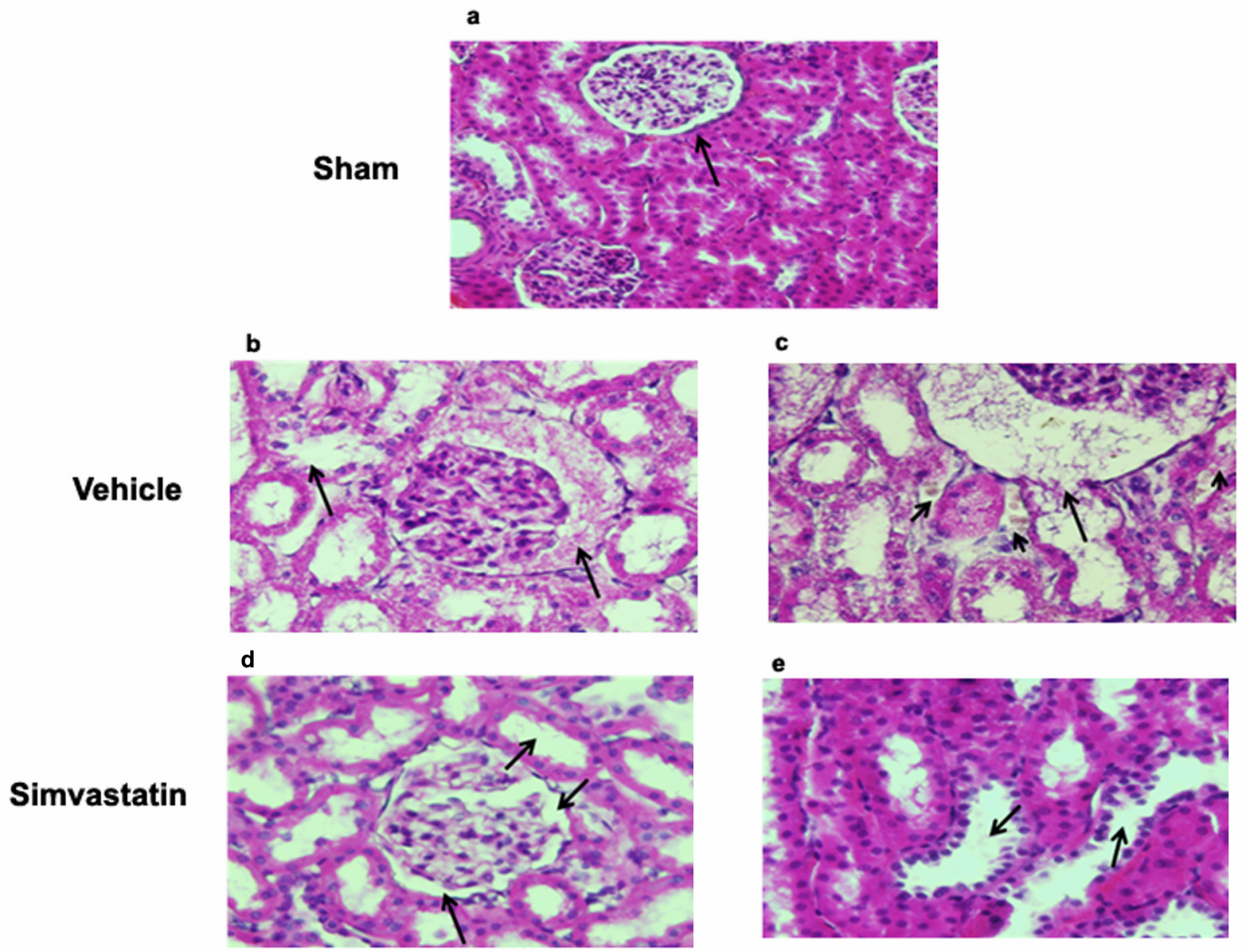
**Simvastatin improves histomorphology of kidney evaluated at 14^th ^day after SCI in rats**. Histomorphology of kidney of sham (a) vehicle (b, c) and simvastatin (d, e) groups was determined by H & E staining. Histology of glomerular complex (arrow) and renal tubules in sham kidney was normal. Bladder hypertrophy results in renal tubular degeneration and glomerular dysfunction (b) and formation of crystals (arrow heads) and degeneration of glomerular wall (arrow) in spinal bladder kidneys (c). Simvastatin treatment significantly reduced the damage observed with renal tubules and glomerular complex (d & e arrows). Photomicrographs are representative of n = 3 in each group. Magnification (a × 400 and b-e × 600).

**Figure 8:**
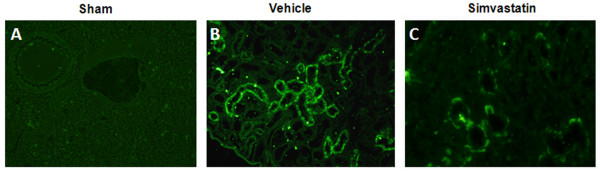
**Simvastatin reduces the expression of caspase-3 in kidney evaluated at 14^th ^day after SCI in rats**. Caspase-3 expression in kidney of sham (A) vehicle (B) and simvastatin groups (C) was determined at 14^th ^day following SCI. Kidney of sham group shows no positive staining of caspase-3. Increased expression of caspase-3 was seen in vehicle group (B, green fluorescence). Simvastatin treatment significantly reduced the expression of caspase-3 (C, green fluorescence). Photomicrographs are representative of n = 3 in each group. Magnification × 400.

The kidney of the sham group showed normal renal tubules and glomerular complexes with the glomerular complex clear and devoid of any filtrate accumulation (Fig 7a). The renal tubules from the vehicle animals showed a remarkable degeneration. An accumulation of filtrate in the glomerular complex was also evident (Fig 7b). In addition, the SCI kidney showed crystals in the intratubular space. Degeneration of the glomerular wall was also seen (Fig 7c). The simvastatin animals showed clear renal tubules (Fig 7d). Glomeruli were without accumulation of any filtrate. Renal tubular degeneration was also reduced (Fig 7e). Next, we examined the expression of caspase-3, an indicator of the apoptotic mechanisms of renal tubular epithelial cell loss (Fig 8). Insignificant staining observed in the sham kidney indicates the absence of caspase-3 activity (Fig 8A). The vehicle kidney showed increased caspase-3-positive renal tubules (Fig 8B), while simvastatin treatment significantly reduced the expression of caspase-3 in renal tubules (Fig 8C).

## Discussion

The present study shows that post-SCI treatment with simvastatin not only reduced the severity of SCI but also attenuated SCI-induced pathological damage to the bladder and kidney in rats. The improvements in voided urine volume, osmolality of urine, and proteinuria correlated with recovery of locomotor function in simvastatin-treated animals.

The post-SCI period is marked by reduced locomotor activity, an increased catabolic rate, and nitrogen loss. In the present study, we observed a 9-fold increase in bladder weight induced by SCI among controls while the animals treated with simvastatin showed only a 2.7-fold increase in bladder weight. The nature of bladder hypertrophy is dependent on specific hypertrophying signals; for example, SCI-induced spinal bladder is characterized by smooth muscle cell hypertrophy of the bladder [9], but alloxan-induced bladder hypertrophy does not involve smooth muscle hypertrophy [33]. The outlet obstruction model induced about a 4-fold increase in rat bladder weight [34] and a 6-fold increase in bladder volume at 3 days after SCI [35]. In addition to weight increase, we also observed increased hyperplasia of the transitional epithelial layer and enlarged tunica adventia in the spinal bladder. The differing relationship observed between the severity of hypertrophy and the hypertrophying signals emphasizes that SCI-induced bladder hypertrophy needs to be studied as an independent pathology. In addition, as SCI patients also show simultaneous contraction of the detrusor and sphincter, resulting in detrusor-sphincter dyssynergia accompanied by urinary retention and increased bladder volume [5], the animal model used in the present study fits well for the purpose of delineating SCI-induced bladder abnormalities as observed in human patients.

The increase in the urine-to-plasma osmolality ratio observed in the present study reflects a reduction in glomerular filtration level [36]. Osmolality reflects the dehydration and hydration status of the individual [37]. Under pathological conditions, low urine osmolality is seen with acute renal failure [38] and nephrotoxicity [39]. Protein excreted in urine reflects the functional status of the kidney [40]. The high urine/plasma protein ratio of SCI animals was significantly reduced by simvastatin treatment. Statins have previously been reported to decrease proteinuria and enhance the glomerular filtration rate in patients with chronic kidney disease [41,42]. Buemi et al have shown fluvastatin to reduce proteinuria in IgA nephropathy patients [43]. Damage to glomeruli and increased infiltration were evident in the hypertrophying bladders of SCI animals, and the degree of damage was less pronounced in the simvastatin group (Fig 7). This reduced glomerular damage may occur through the direct effect of statins on mesangial cells [44]. Observed recovery of the spinal reflex of bladder following SCI has varied from 7 [9] to 14 days [4]; however, despite recovery of spinal reflex, complete voiding efficiency was not recovered [45]. The increased pressure due to retention of a large volume of urine in the bladder forces the urine back into the ureter and hilus of the kidney, thus disturbing the renal medulla [46] and the cortico-medullary interstitial gradient, affecting urine concentration [47]. Hence, with increased urine retention and proteinuria, we anticipated that bladder hypertrophy would affect normal renal architecture. Histological and immunofluorescence studies with kidney (Fig 7 &8) supported this assumption.

Degeneration of plaques as observed in spinal bladder has serious consequences because the urothelium, in addition to serving as a passive barrier between urine and detrusor muscle, is involved in antigen presentation, micturition reflex, and inflammatory regulation [48]. Simvastatin treatment preserved the membrane plaques and protected the underlying urothelium. The simvastatin group showed a decreased level of crystals in the kidney (Fig 7d, e). This observation is interesting as humans and rats have similar crystal-clearing mechanisms [49]. In addition, statins have already been shown to inhibit renal crystal formation in rats [24]. Renal inflammation is one of the factors responsible for stone formation [50]. Anti-inflammatory and neuroprotective properties of statins are now well established [18,29,51]. Therefore, the reduced number of caspase 3 positive cells in simvastatin-treated rats supports earlier studies of reduced cell death by rosuvastatin [52] and atorvastatin (22). However, the link between locomotion recovery and damage to bladder and kidney following SCI is not clear. Our data indicate that the degree of bladder/kidney dysfunction and their recovery are dependent on the severity of injury and the associated myelin/white matter loss at the injury site [4]. Simvastatin-mediated recovery of bladder dysfunction may be due, at least in part, to enhanced neuroprotection within the spinal cord. Increased recovery of locomotor behavior and improved renal/bladder functions in simvastatin-treated animals supported the overall efficacy of simvastatin therapy in SCI. In addition, atorvastatin was also reported to attenuate SCI-induced blood spinal cord barrier (BSCB) leakage [28]. We have used several statins, including lovastatin, simvastatin and atorvastatin, interchangeably with comparable effects in animal models of neuroinflammatory diseases, suggesting a class effect of HMG-CoA reductase inhibitors. They exert significant effects on oxidative stress and inflammation within a few hours. However, pleiotropic effects on endothelial functions seem to appear earlier for simvastatin than for atorvastatin. Although the half life of atorvastatin is longer (~7 h) [53] than simvastatin (~4.5 h) [54], simvastatin's lipophilic qualities determines its superior tissue accessibility, thus making it especially promising for longer treatment regimens when inflammation is diminishing, but repair and recovery are in progress. Therefore, simvastatin (5 mg/kg) was elected in this study. The dose 5 mg/kg was based on studies with atorvastatin in rat SCI models [28,55]. Lower dose such as 0.5 mg/kg of simvastatin significantly improves functional outcome in a rat model of traumatic brain injury [56]. Higher dose of simvastatin (20 mg/kg) is reported to be toxic in experimental SCI studies [57].

The early recovery of a bladder contraction reflex in simvastatin-treated animals may also be due to the effect of the drug on the spinal bladder itself. For example, induction of inositol 1,4,5 triphosphate (IP3) stimulates the initial contractile response of bladder smooth muscle [58], and simvastatin has been shown to increase the cellular level of IP3 [59]. Nevertheless, bladder hypertrophy involves several factors, including M2 receptors [60], neurotransmitters like glutamate [61], transcriptional factors like STAT3 [6], nerve growth factor [62], intracellular calcium [34], transforming growth factor beta [63], basic fibroblast growth factor [7], and protein kinase C [64]. Therefore, the exact signaling cascade involved in simvastatin-mediated bladder and renal functional recovery remains to be elucidated. Nonetheless, the overall beneficial effects of statins indicate their potential for effecting quick clinical benefits in aiding tissue repair.

## Conclusions

The present study suggests that treating SCI patients with simvastatin may help to ameliorate bladder and renal dysfunction in addition to providing recovery of locomotor functions.

## List of Abbreviations

BAMG: bladder acellular matrix graft; BBB: Basso Beattie Bresnahan; BOO: bladder outlet obstruction; BSCB: blood spinal cord barrier; CCI: controlled contusion injury; EAE: experimental autoimmune encephalomyelitis; EUS: external urethral sphincter; FDA: food and drug administration; FES: functional electrical stimulation; H&E: hematoxylin and eosin; IACUC: institutional animal care and use committee; IP-3: inositol 1,4,5 triphosphate; PBS: phosphate buffered saline; SCI: spinal cord injury; SD: standard deviation; STAT3: signal transducer and activator of transcription 3.

## Competing interests

The authors declare that they have no competing interests.

## Authors' contributions

This study is based on an original idea of AS, MK and IS. MK and AS wrote the manuscript. AS, PCC, RKD, MB carried out animal and biochemical studies. AS, MM, AGC, BRS, TCS performed histochemical studies. JKO critically examined renal and bladder studies and corrected the manuscript. All authors have read and approved the manuscript.
